# Change in functional profile after lumbar spinal surgery: a register-based study among 1,451 patients

**DOI:** 10.2340/17453674.2025.42850

**Published:** 2025-02-14

**Authors:** Konsta K J KOIVUNEN, Sara S WIDBOM-KOLHANEN, Katri I PERNAA, Jari P A AROKOSKI, Mikhail SALTYCHEV

**Affiliations:** 1 Department of Clinical Medicine, Turku University Hospital and University of Turku, Turku; 2 Department of Surgery, Satasairaala Hospital, Pori; 3 Department of Orthopedics, Turku University Hospital and University of Turku, Turku; 4 Department of Physical and Rehabilitation Medicine, Helsinki University Hospital and Helsinki University, Turku; 5 Department of Physical and Rehabilitation Medicine, Turku University Hospital and University of Turku, Turku, Finland

## Abstract

**Background and purpose:**

The Oswestry Disability Index has usually only been used as a composite score but, according to previous studies, disability caused by back pain may be too broad a concept to be explained by a single number. We aimed to analyze changes in each ODI item’s score from preoperative to 3, 12, and 24 months after surgery by creating a functional profile.

**Methods:**

This was a register-based study of 1,451 patients undergoing lumbar spinal surgery between 2018 and 2021. The patients responded to a repeated survey preoperatively and 3, 12, and 24 months after surgery. The significance of change in the ODI items’ scores was assessed by a symmetry test.

**Results:**

All the ODI items’ scores and total score improved between baseline and 3-month follow-up (P < 0.001). The magnitude of this improvement varied across different items. After 3 months, no significant change was seen for most of the items.

**Conclusion:**

During a postoperative 2-year follow-up, individual items of the ODI demonstrated changes of different magnitude. The results imply that the use of a single composite score of the ODI might be insufficient to describe changes in functioning among patients undergoing lumbar spinal surgery. Instead, in some situations, creating a functional profile based on the scores from individual items may be a better solution to describe the changes in disability level.

Different patient-reported outcome measures (PROMs) have been proposed to assess changes in functioning after spinal surgery [1]. Most of them produce single composite scores, which are usually simply arithmetic sums of item scores. The 10-item Oswestry Disability Index (ODI) is one such scale. The ODI has been a gold standard, a well-validated and highly reliable tool, to evaluate disability caused by low back pain related to different lumbar spinal conditions [2,3].

A single number may hardly define such complex concepts as functioning or disability [4]. Some PROMs did recognize this weakness, suggesting that a composite score should be accompanied by a disability profile, which describes different domains of functioning [5]. While functional profiles have not been used widely among patients with back pain, knowledge concerning restrictions in each domain of the ODI may be of great value when planning or executing rehabilitation or treatment intervention. The interpretation of a graphically presented profile is an easy and intuitive process not requiring any substantial training. Certainly, the use of such profiles should be supported by necessary software solutions, preferably integrated into existing electronic patient records or other similar registers.

When the goal is to describe the average situation in a larger group, the composite score is certainly a more reasonable option. However, at an individual level, a functional profile may be a better option, especially if the expected change in disability is modest and probably depends on a change in only one or a few areas of functioning. An example can be a relatively short treatment or rehabilitation intervention.

In 2001, the WHO introduced the International Classification of Functioning, Disability and Health (ICF), which represents a biopsychosocial model of functioning [6]. The usefulness and need for the ICF-based functional profiles, when assessing disability caused by back pain in different settings, have been stated by multiple studies [7-9]. From the ICF’s point of view, functioning is a very broad concept that describes the effect of damage to a body part on the function (activity) of this body part, participation on a social level, and the external factors that help or hinder activity and participation. This complex approach does not favor defining functional impairment through a single rough number. That is why the WHO and its collaborating teams have offered more detailed ways to describe functional capacity. Examples can be the WHO’s generic ICF checklist or the comprehensive and brief core sets for low back pain developed by the Research Branch [10-12].

We aimed to analyze changes in each ODI item’s score from preoperatively to 3, 12, and 24 months after surgery by creating a functional profile.

## Methods

### Study design

This was a retrospective study based on data from patients who were undergoing a lumbar spinal surgery of any kind between June 21, 2018 and August 17, 2021. A patient was included if the procedure code was one of the following: ABC36, ABC56, ABC66, NAG61, NAG62, NAG63, NAG66, and NAG67 according to the Nordic Classification of Surgical Procedures (NCSP), version 1.15. All patients who have undergone more than 1 procedure during a follow-up were excluded. This study was reported according to the STROBE guidelines.

### Study setting

The data was obtained from the ongoing study among patients undergoing spinal surgery (CTL Study) in Turku University Hospital located in south-west Finland. The register has not been explicitly validated. The patients responded to repeated surveys: ≤ 2 months before the surgery (baseline wave #1); 2–4 months after the surgery (wave #2); 11–13 months after the surgery (wave #3); and 23–25 months after the surgery (wave #4). The survey contained questions on demographics and the severity of disability. The register that provided the data was part of the electronic patient record system used by a university hospital. The patients received a protected link to a questionnaire. Some of the data was added by physicians or nursing personnel, and some was extracted from the other information available through patient records. The researchers were unable to affect the process of data gathering.

### Independent variables

Age was defined in full years at the time of surgery. Body mass index (BMI) was self-reported by the patients and defined as a bodyweight in kg divided by squared height in meters.

The duration of pain was defined in years at the time of surgery and dichotomized as ≤ 1 year vs > 1 year. Back and leg pain intensity was assessed by using a visual analogue scale (VAS) of 0 to 100 points with 0 indicating “no pain” and 100 indicating “worst possible pain.” While there was no explicit data on the occupational status of the participants, the age of statutory retirement in Finland is around 65 years. At this age, people start receiving a pension regardless of whether they still work for a certain amount of time or not. It is not possible to determine these possible additional work activities during statutory retirement.

### Dependent variables (outcomes)

The ODI is a questionnaire containing 10 items defining restrictions in daily functioning caused by low back pain. Each item is assessed on a 6-level ordinal scale with 0 describing “no limitation” and 5 describing “extreme limitation or inability to function.” The total score is a percentage calculated as a sum of all answers divided by 50 (the highest possible score) and multiplied by 100. The equation is adjusted when the responses to 1 or more items are missing. A score of 0 points represents the highest possible level of functioning and independence while a score of 100 points represents the lowest level of functioning with total dependence.

### Statistics

The descriptive statistics were reported as absolute numbers and percentage, as means and standard deviations (SD), or as medians and interquartile ranges (IQR), when appropriate. To test the significance of before–after change in the ODI scores, a symmetry test was used reporting chi-square statistics and P values for an asymptotic symmetry test and a Stuart–Maxwell test for marginal homogeneity (a general test for matched-pair data with polytomous responses in biomedical research) [13]. Due to abnormal distribution, the significance of the change in the ODI total score was assessed by using median regression. The level of significance was set at < 0.05. To assess the change in items’ scores, a quantile regression (also known as least absolute value, minimum absolute deviation, or minimum L1-norm value) was employed. To calculate 95% CIs for medians, we used a binomial method for obtaining confidence intervals that makes no assumptions concerning the underlying distribution of the variable. The conservative confidence interval was obtained, forcing the confidence limits to fall exactly on sample values. All the data analyses were performed utilizing Stata 18 (StataCorp LLC, College Station, TX, USA).

### Ethics, data sharing plan, funding, use of AI, and disclosures

According to the ethics board of the university hospital district, a register-based study employing hospital electronic patient records does not need explicit approval or individual informed consent. The data had been delivered to the research team by the hospital district IT office without any identifiable information. The data is available on a reasonable request from MS (mikhail.saltychev@gmail.com). The research followed the Declaration of Helsinki. Due to the retrospective register-based nature of the study, neither patients nor members of the public were included in the implementation of the study. No funding was received. AI tools were not used. The authors declare no conflicts of interests. Complete disclosure of interest forms according to ICMJE are available on the article page, doi: 10.2340/17453674.2025.42850

## Results

**Figure 1:**
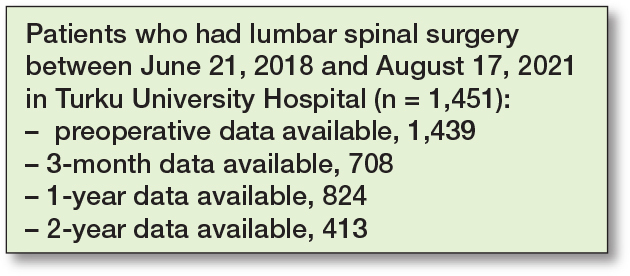
Patient flowchart. Numbers are shown for the ODI item #1. Numbers for different items were slightly varying (see Table 2).

The preoperative surveys were completed by 1,451 patients with a mean age of 67 years (Figure 1). Of these, 793 (55%) were women and 658 (45%) were men (Table 1). The mean BMI was 29. Among the patients, 567 (39%) reported pain for ≤ 1 year and 884 (61%) had experienced pain for > 1 year before surgery. The most frequent reasons for surgery were “M48 Spondylopathies” 862 (59%), “M43 Deforming dorsopathies” 224 (15%), and “M47 Spondylosis” 114 (8%). The most frequent surgical techniques were “ABC36 Decompression of lumbar nerve roots” 418 (29%), “NAG62 Posterior fusion with fixation, 2–3 vertebrae” 412 (28%), and “ABC56 Decompression of spinal canal and nerve roots” 370 (26%).

**Table 1:** Baseline characteristics of the study population (N = 1,451). Values are count (%) unless otherwise specified

Variable	Mean or n (SD or %)
Age, years, mean (SD)	66.9 (12.1)
Body mass index, mean (SD)	28.9 (4.9)
Back pain intensity, mean (SD)	58.9 (26.8)
Leg pain intensity, mean (SD)	63.6 (26.3)
Oswestry Disability Index total score, mean (SD)	41.9 (16.9)
Sex	
Men	658 (45)
Women	793 (55)
Pain duration before surgery	
≤ 1 year	567 (39)
> 1 year	884 (61)
Surgery codes **^a^**	
Decompression (ABC36, ABC56, ABC66)	856 (59)
Fusion (NAG62, NAG63, NAG66)	573 (39)
Others	22 (1.5)
Main diagnoses **^b^**	
Spondylopathies (M48)	862 (59)
Deforming dorsopathies (M43)	224 (15)
Spondylosis (M47)	114 (7.9)
Nerve root and plexus compressions in diseases classified elsewhere (G55)	80 (5.5)
Thoracic, thoracolumbar, and lumbosacral intervertebral disc disorders (M51)	64 (4.4)
Scoliosis (M41)	37 (2.6)
Bursopathies (M71)	23 (1.6)
Others	47 (3.3)

a Nordic Classification of Surgical Procedures

b International Classification of Diseases ICD-10

The rates of dropouts between repeated measures were substantial, especially between the baseline and the first postoperative follow-up at 3 months (Table 2).

**Table 2:** Oswestry Disability Index items’ scores at different time points

Item/time-point	Median	IQR	CI	n
Item #1 Pain intensity				
Baseline	2	2–3	2–3	1,439
3 months	1	1–2	1–1	708
1 year	1	0–2	1–1	824
2 years	1	0–2	1–1	413
Item #2 Personal care				
Baseline	1	0–2	1–1	1,443
3 months	0	0–1	0–0	705
1 year	0	0–1	0–0	820
2 years	0	0–1	0–0	414
Item #3 Lifting				
Baseline	3	2–4	3–3	1,437
3 months	2	1–3	1–2	696
1 year	1	0–3	1–1	820
2 years	2	1–3	1–2	412
Item #4 Walking				
Baseline	2	1–3	2–2	1,438
3 months	1	0–1	1–1	698
1 year	1	0–2	1–1	822
2 years	1	0–2	0–1	412
Item #5 Sitting				
Baseline	2	1–2	2–2	1,437
3 months	1	0–2	1–1	705
1 year	1	0–2	1–1	823
2 years	1	0–2	1–1	413
Item #6 Standing				
Baseline	3	2–4	3–3	1,432
3 months	1	0–3	1–1	700
1 year	1	0–3	1–2	822
2 years	1	0–3	1–2	413
Item #7 Sleeping				
Baseline	1	1–2	1–1	1,435
3 months	1	0–1	1–1	704
1 year	1	0–1	1–1	824
2 years	1	0–1	1–1	414
Item #8 Sex life				
Baseline	1	0–4	1–2	1,105
3 months	0	0–1	0–0	552
1 year	0	0–1	0–0	631
2 years	0	0–1	0–0	326
Item #9 Social life				
Baseline	3	2–3	2–3	1,431
3 months	0	0–2	0–0	695
1 year	0	0–2	0–0	810
2 years	0	0–2	0–1	407
Item #10 Travelling				
Baseline	2	1–3	2–2	1,403
3 months	1	0–1	1–1	678
1 year	1	0–1	1–1	804
2 years	1	0–2	1–1	399

CI: 95% confidence interval; IQR: interquartile range.

### Outcomes

**Figure 2:**
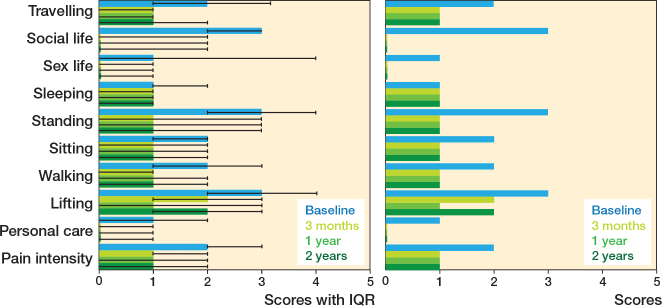
Change in profile of functioning based on Oswestry Disability Index presented as a bar chart with median and interquartile range (IQR) for scientific use (left panel) and simplified version without IQR for clinical use.

There were significant changes in the ODI items’ scores 3 months after surgery, but not after that (with a few exception) (Table 3). Most of the ODI items demonstrated considerable improvement from the baseline (Figure 2). Improvement in item #7 “sleeping” was minor whereas “lifting” showed significant initial improvement but worsened slightly between 1 and 2 years after the surgery. “Standing” and “social life” showed the greatest improvement from the baseline (Figure 2). The median ODI total score was 40 (IQR 30–54) points at baseline, 18 (IQR 8–30) points 3 months after surgery, 18 (IQR 6–30) points at 1 year, and 20 (IQR 6–34) points at 2 years after surgery. The improvement in the ODI total score was significant, with slope of –0.93 (SE 0.41, CI –10.1 to –8.5, pseudo R2 0.12) (Table 4).

**Table 3:** Symmetry test for change in the Oswestry Disability Index items’ scores

Change	Test	Chi square	df	P value	n
Item #1 Pain intensity					
3 months vs baseline	AS	393	13	<0.001	702
	MH	367	5	<0.001	702
1 year vs 3 months	AS	17	10	0.06	426
	MH	13	5	0.02	426
2 years vs 1 year	AS	12	11	0.3	346
	MH	7.9	5	0.2	346
Item #2 Personal care					
3 months vs baseline	AS	256	13	<0.001	701
	MH	249	5	<0.001	701
1 year vs 3 months	AS	12	11	0.4	424
	MH	5.1	5	0.4	424
2 years vs 1 year	AS	7.8	9	0.6	346
	MH	7.0	4	0.1	346
Item #3 Lifting					
3 months vs baseline	AS	167	15	<0.001	692
	MH	153	5	<0.001	692
1 year vs 3 months	AS	26	14	0.02	414
	MH	20	5	0.001	414
2 years vs 1 year	AS	22	14	0.08	345
	MH	7.6	5	0.2	345
Item #4 Walking					
3 months vs baseline	AS	350	14	<0.001	692
	MH	315	5	<0.001	692
1 year vs 3 months	AS	9.1	11	0.6	421
	MH	3.8	5	0.6	421
2 years vs 1 year	AS	14	10	0.2	347
	MH	8.3	4	0.08	347
Item #5 Sitting					
3 months vs baseline	AS	149	12	<0.001	699
	MH	141	5	<0.001	699
1 year vs 3 months	AS	12	9	0.2	424
	MH	9.1	4	0.06	424
2 years vs 1 year	AS	21	10	0.02	347
	MH	15	5	0.009	347
Item #6 Standing					
3 months vs baseline	AS	381	15	<0.001	693
	MH	333	5	<0.001	693
1 year vs 3 months	AS	9.4	13	0.7	420
	MH	3.4	5	0.6	420
2 years vs 1 year	AS	18	12	0.1	349
	MH	9.0	5	0.1	349
Item #7 Sleeping					
3 months vs baseline	AS	322	14	<0.001	702
	MH	299	5	<0.001	702
1 year vs 3 months	AS	14	10	0.2	420
	MH	7.2	5	0.2	420
2 years vs 1 year	AS	18	12	0.1	349
	MH	6.3	5	0.3	349
Item #8 Sex life					
3 months vs baseline	AS	232	15	<0.001	518
	MH	201	5	<0.001	518
1 year vs 3 months	AS	9.7	14	0.8	315
	MH	3.8	5	0.6	315
2 years vs 1 year	AS	7.8	13	0.9	257
	MH	0.34	5	1.0	257
Item #9 Social life					
3 months vs baseline	AS	403	15	<0.001	689
	MH	388	5	<0.001	689
1 year vs 3 months	AS	12	11	0.4	412
	MH	4.7	5	0.5	412
2 years vs 1 year	AS	18	10	0.06	337
	MH	15	5	0.01	337
Item #10 Travelling					
3 months vs baseline	AS	268	15	<0.001	666
	MH	251	5	<0.001	666
1 year vs 3 months	AS	24	12	0.02	403
	MH	15	5	0.01	403
2 years vs 1 year	AS	9.6	11	0.6	332
	MH	4.3	5	0.5	332

AS: asymptotic symmetry test; MH: Stuart–Maxwell test for marginal homogeneity; df: degrees of freedom.

**Table 4:** Change in items’ scores from preoperative to the longest available follow-up (quantile regression)

Items	Regression coefficient	Standard error	CI
Item #1 Pain intensity	–0.50	0.03	–0.56 to –0.44
Item #2 Personal care	–0.33	0.01	–0.35 to –0.31
Item #3 Lifting	–0.67	0.03	–0.73 to –0.61
Item #4 Walking	–0.67	0.03	–0.73 to –0.61
Item #5 Sitting	–0.50	0.04	–0.58 to –0.42
Item #6 Standing	–0.67	0.03	–0.73 to –0.60
Item #7 Sleeping **^a^**	–	–	–
Item #8 Sex life	–0.33	0.02	–0.37 to –0.29
Item #9 Social life	–0.67	0.04	–0.75 to –0.59
Item #10 Travelling	–0.50	0.03	–0.56 to –0.44
Total score	–9.33	0.41	–10 to –8.5

a Model did not achieve meaningful results.

CI: 95% confidence interval.

## Discussion

This observational register-based study examined patients undergoing different lumbar spinal surgical procedures. We aimed to analyze changes in each ODI item’s score from preoperative to 3, 12, and 24 months after surgery by creating a functional profile. Most of the ODI items, as well as the composite score, showed significant improvement from the baseline through the entire 2-year follow-up. However, the magnitude of these improvements varied across different items. The only unchanged item was “sleeping.” The most substantial changes were seen in “standing” and “social life.”

These results were in line with previous research. In 2012, Djurasovic et al. have reported that “social life” was one of the most improved items in patients undergoing lumbar fusion [14]. In 2018, Murphy et al. studied patients undergoing surgery due to lumbar spondylolisthesis, reporting “standing” as the most improved and “sleeping” as the least improved items [15]. As standing is required for a variety of other activities (e.g., walking or travelling), it is easy to understand why “standing” is strongly linked to overall functioning [16]. Participation in social life has previously been directly linked to physical functioning, which may explain the importance of “social life” to overall functioning in the studied cohort [17]. Sleep difficulties have previously shown great persistence over time. It has been suggested that sleep patterns are an intrinsic trait, usually only weakly altered by aging or by external factors [18,19].

While the ICF has proved its usefulness when evaluating functioning of patients with low back pain [7,20,21], the use of ICF-oriented functional profiles in patients undergoing lumbar spinal surgery has been scarce [8]. The present findings support the hypothesis that the composite scores of PROMs may describe functioning of an individual patient or a small group imprecisely. While composite scores may play an important role when assessing disability on a population level, they may fail to comprehensively describe disability on an individual level. The potential benefits of using profiles along with total scores or instead of them have been suggested for the assessment of patients with low back pain as well as other health conditions [22,23]. The use of a functional profile in addition to a composite score may provide valuable information on functional restrictions experienced by a patient. Such knowledge may be crucial when planning treatment or rehabilitation, or evaluating the results of interventions. Graphical presentation of the functional profile provides a convenient way to easily assess limitations across different functional domains. This may be of help for both medical professionals and patients (as well as for their caregivers). Observing improvements in their own functional profiles may encourage patients to improve their commitment to a post-surgery rehabilitation plan.

The generalizability of these results might be affected by several factors. The study was conducted in a single highly specialized university-based spine clinic and the changes in disability level might be different in a general hospital. Also, this study material included several different surgical techniques. It is possible that changes in functioning are of different magnitudes or even in different directions in diverse disease groups and in dissimilar surgical procedures. Most of the patients were close to 70 years of age, which may affect the inferences regarding other age groups. The distribution of diagnoses and reasons for spine surgery may fluctuate in different age groups. Additionally, some of the ODI items may be of different importance for people in different age groups, e.g., “work” or “sex life.” Data on the fitness of the patients, their marital status, and smoking status prior to surgery as well as the length of stay in a hospital was unavailable. Being married and non-smoking have been associated with a greater benefit from surgery in previous studies [24,25]. Also, higher levels of disability have been associated with low levels of physical activity [26]. A higher ODI score has also been associated with a longer stay in a hospital. All these missing data might affect the magnitude of changes in disability level after surgery. The missing data was not controlled by the research group. Thus, there was uncertainty regarding potential differences between respondents and non-respondents. Also, the results showed considerable attrition over the period of follow-up, which could affect the results. However, it has to be noted that this weakness could not affect the main goal of the study: demonstrating the use of the ODI as a functional profile instead of, or in addition to, the composite score.

### Conclusion

The composite score and the scores of most of the items of the ODI improved significantly during a 2-year follow-up among patients undergoing different lumbar spinal surgical procedures. The ODI items showed, however, different magnitude of improvement. The results imply that the use of a single composite ODI score might be insufficient to describe changes in functioning among patients undergoing lumbar spinal surgery. Instead, in some situations, creating a functional profile based on the scores from individual items may be a better solution to describe the changes in disability level.

## Notes

Authors KK, KP, and MS have substantially contributed to the conception and design of the work and to the analysis and interpretation of data for the work. KP and MS have been responsible for the acquisition of the data. KK has been responsible for drafting the work, SWK, KP, JA, and MS have revised it critically for important intellectual content. All of the authors have approved the final version to be published as well as the order of the authors.

Handling co-editors: Paul Gerdhem and Philippe Wagner

*Acta* thanks Björn Knutsson and Freyr Gauti Sigmundsson for help with peer review of this manuscript.
